# Regulation of VCP/p97 demonstrates the critical balance between cell death and epithelial-mesenchymal transition (EMT) downstream of ER stress

**DOI:** 10.18632/oncotarget.3918

**Published:** 2015-04-23

**Authors:** Parag P. Shah, Levi J. Beverly

**Affiliations:** ^1^ James Graham Brown Cancer Center, University of Louisville, Louisville, KY, USA; ^2^ Department of Medicine, Division of Hematology and Oncology, University of Louisville School of Medicine, Louisville, KY, USA; ^3^ Department of Pharmacology and Toxicology, University of Louisville School of Medicine, Louisville, KY, USA

**Keywords:** ER stress, p97, ERAD, EMT, migration, invasion, metastasis

## Abstract

Valosin-containing protein (VCP), also called p97, is a AAA+ ATPase that has been shown to be involved in endoplasmic reticulum-associated protein degradation (ERAD), mitochondria quality control and vesicle transport. We and others have previously found that disruption of VCP is sufficient to cause endoplasmic reticulum (ER) stress. We observed that induction of ER stress either following siRNA mediated loss of VCP or inhibition of VCP with eeyarestatin I potently activates an EMT-like state in cells. Interestingly, both ER stress and EMT are reversible events. Further, brief treatment of cells with eeyarestatin I increases EMT markers, and migratory and invasive properties of lung cancer cells. By examining primary lung adenocarcinoma patient samples we find that the VCP locus is heterozygously lost in nearly half of lung adenocarcinomas and VCP protein expression is decreased in nearly all primary lung tumors. Further, primary lung adenocarcinomas have increased ER stress and EMT markers. These observations have potential clinical relevance because increased ER stress and EMT markers are known to contribute to chemoresistance and poor survival of patients with lung adenocarcinoma.

## INTRODUCTION

The endoplasmic reticulum (ER) is the main eukaryotic organelle involved in protein synthesis and protein export [1]. Unwanted or misfolded proteins in the ER are exported into the cytoplasm and degraded by the proteasome through the ER associated protein degradation pathway (ERAD). Disturbances in ERAD result in ER stress, which in turn signals the unfolded protein response (UPR) [2, 3]. UPR can result from accumulation of misfolded proteins within the ER. In order to alleviate this burden, the UPR pathway signals through IRE-1, ATF6 and PERK to promote the activation of genes required to restore ER homeostasis, including components of ERAD [4, 5]. If cells cannot recover from excessive ER stress they will undergo apoptosis [6, 7]. In addition, previous studies have suggested that, along with apoptosis, ER stress may induce pathways involved in cell differentiation, and morphological changes in order to deal with the consequences of ER stress.

Valosin-containing protein (p97/VCP) is responsible for maintaining the integrity of ER and has been shown to be involved in ERAD [8]. VCP is also involved in a wide variety of cellular processes including ubiquitin-dependent protein degradation, stress responses and programmed cell death [9]. Moreover, VCP has been shown to be involved in pathogenesis of wide variety of neurodegenerative diseases including Alzheimer's disease, Parkinson's disease and Amyotrophic Lateral Sclerosis (ALS) [10, 11], possibly via regulation of ER homeostasis. It is thought that ER stress plays a role in a wide variety of diseases, including lung cancer. However, the mechanism by which ER stress contributes to tumor initiation, progression, and metastasis is not clear.

Epithelial-mesenchymal transition (EMT) was identified as an important and crucial process involved in embryonic development, has been shown to be a key step during tumor progression and metastasis [12]. EMT has been described as an orchestrated event during which polarized epithelial cells differentiate into more motile and migratory mesenchymal-like cells [13]. Loss of epithelial markers, such as E-cadherin, and gain of mesenchymal markers, such as Vimentin, are the hallmark of EMT. Several factors including Snail, Slug, the zinc-finger factors protein family 1 and 2 (ZEB1/ZEB2) and Twist directly regulate the transcription of genes involved in EMT [14].

Recent studies have demonstrated that ER stress induces EMT in different cellular systems [15, 16]. In the study by Tanjore et al., ER stress activated numerous pathways including MAPK, Smad and β-catenin in alveolar epithelial cells. In addition, they showed that EMT markers were induced following ER stress downstream of Src kinase signaling [17]. In the present study, we show that loss of VCP induces ER stress and EMT. Sustained inhibition or loss of VCP eventually leads to cell death, presumably because the cells are not able to withstand the level of ER stress that results from loss of VCP function. However, before the cells succumb to ER stress induced cell death, the cells upregulate an EMT-like state. Interestingly, by using Eeyarestatin I (EerI), a known VCP inhibitor, we find that ER stress and EMT are reversible events. In fact, restoration of VCP function, by withdrawing the inhibitor, allows cells to recover from the ER stress but increases the migration and invasion of cells. Examination of primary clinical samples demonstrated that lung adenocarcinomas have decreased VCP expression, increased expression of ER stress markers and activation of EMT markers when compared to match normal adjacent tissues. These observations have potential clinical relevance because increased expression of ER stress markers and activation of EMT markers along with decreased expression of VCP in lung adenocarcinoma patients may be helpful for understanding the mechanism involved in ER stress induced EMT and designing the better therapeutic strategies.

## RESULTS

### Loss of VCP induces ER stress

**Figure 1:**
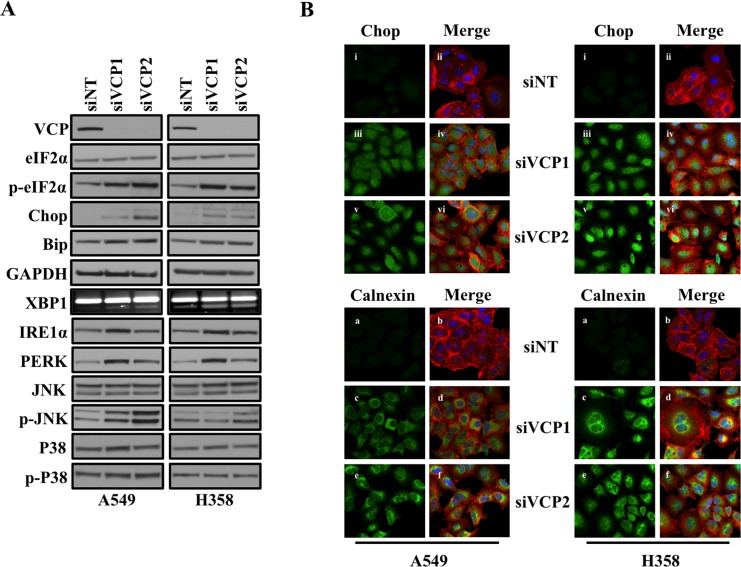
Loss of VCP induces ER stress **A.** Western blot analysis of different proteins involved in unfolded protein response and ER stress response. Cells were transfected with either non targeting (siNT) siRNA or two different siRNAs targeting VCP (siVCP1 and siVCP2). After 72 hrs of transfection, cells were harvested and analyzed for protein expression. **B.** Fluorescence staining for Chop and Calnexin in A549 and H358 cells. After 24 hrs of transfection either with non-targeting siRNA (siNT) or with siRNAs targeting VCP (siVCP1 or siVCP2) cells were trypsinized and plated on chamber slides. After 48 hours cells were fixed and stained for Chop and Calnexin. i, iii and v: Chop was detected using Alexa Fluor 488 rabbit anti-mouse IgG (green). ii, iv and vi: overlay of respective Chop and F-actin (Alexa Fluor 568 Phalloidin; red) staining with DAPI counter stain. a, c and e: Calnexin was detected using Alexa Fluor 488 goat anti-rabbit IgG (green). b, d and f: overlay of respective Calnexin and F-actin (Alexa Fluor 568 Phalloidin; red) staining with DAPI counter stain.

VCP has been shown to be an important regulator of ER homeostasis, as it is known to mediate the ER-associated protein degradation pathway (ERAD) [8, 9]. Consistent with these studies, we found that loss of VCP induced ER stress and activated an unfolded protein response (UPR) signaling cascade (Figure 1). Loss of VCP resulted in significant increase in expression of Chop and Bip, which are involved in proper folding of misfolded proteins during ER stress, and increased phosphorylation of downstream eIF2α, which leads to overall decrease in protein translation (Figure 1A). As further confirmation that VCP loss induced UPR, we showed that loss of VCP activated downstream signaling cascade including IRE1α, PERK, p38 and JNK [18] (Figure 1A). We also observed splicing of the mRNA encoding XBP-1 following loss of VCP. Increased in Chop and Calnexin expressions, following loss of VCP in lung adenocarcinoma cells, were also confirmed by immunofluorescence staining (Figure 1B). These data clearly show that inhibition of VCP function causes loss of ER homeostasis.

### VCP loss induces EMT in A549, H358 and HPLD-1 cells

**Figure 2:**
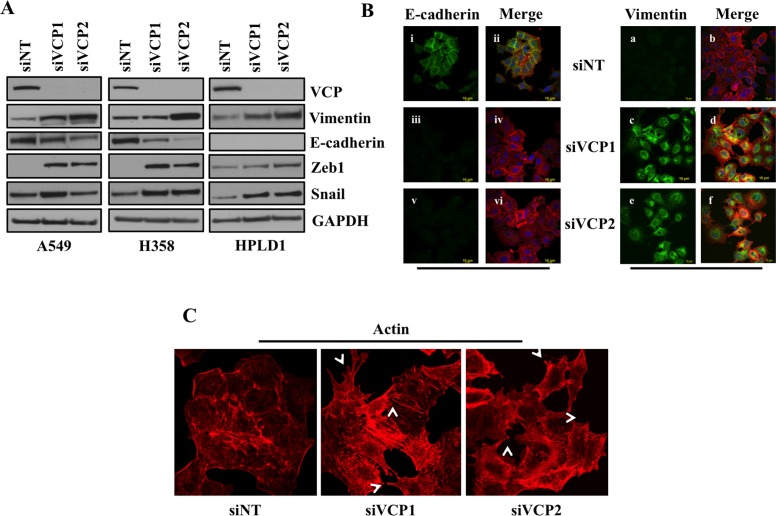
Loss of VCP induces EMT **A.** 72 hours after transfection of A549, H358 or immortalized human bronchiolar HPLD-1 cells with siRNA against VCP or non-targeting cell lysates were prepared and western blot analysis of EMT markers Vimentin and E-cadherin was performed. **B.** Fluorescence staining for E-cadherin and Vimentin in A549 cells. After 24 hrs of transfection either with non-targeting siRNA (siNT) or with siRNAs targeting VCP (siVCP1 or siVCP2) cells were trypsinized and plated on chamber slides and stained for EMT markers. i, iii and v: E-cadherin was detected using Alexa Fluor 488 goat anti-rabbit IgG (green). ii, iv and vi: overlay of respective E-cadherin and F-actin (Alexa Fluor 568 Phalloidin; red) staining with DAPI counter stain. a, c and e: Vimentin was detected using Alexa Fluor 488 goat anti-rabbit IgG (green). b, d and f: overlay of respective Vimentin and F-actin (Alexa Fluor 568 Phalloidin; red) staining with DAPI counter stain. **C.** A549 cells were prepared as described in B and F-actin was detected with Alexa Fluor 568 Phalloidin (red). Re-organization of actin cytoskeleton through destruction and cellular protrusion formation is indicated by arrows.

Previous work has suggested that ER stress can activate an EMT-like state in some cell types. Thus, we determined whether loss of VCP in lung adenocarcinoma cells induces EMT by changing the expression levels of key EMT regulators. We first performed western blot analysis for E-cadherin, Vimentin, Snail and Zeb1 following knockdown of VCP. We found that loss of VCP in both A549 and H358 cells caused decreased expression of epithelial marker E-cadherin and increased expression of mesenchymal proteins Snail, Vimentin and Zeb1 (Figure 2A). Interestingly, similarity in results was observed in case of immortalized human bronchiolar HPLD-1 cells [19]. Furthermore, induction of EMT by loss of VCP was confirmed by performing immunofluorescence staining for E-cadherin and Vimentin (Figure 2B). There was a significant decrease in expression of E-cadherin in A549 cell after siRNA mediated loss of VCP (Figure 2B panels iii and v) and expression of Vimentin in these cells was significantly increased (Figure 2B panels c and e). Further, EMT is often characterized by actin cytoskeleton reorganization and formation of cellular protrusion, which were observed following VCP loss (Figure 2C). Thus, VCP is critical to not only maintain ER homeostasis, but to also repress mesenchymal transition of epithelial cells.

### Prolong VCP loss causes cell death

**Figure 3:**
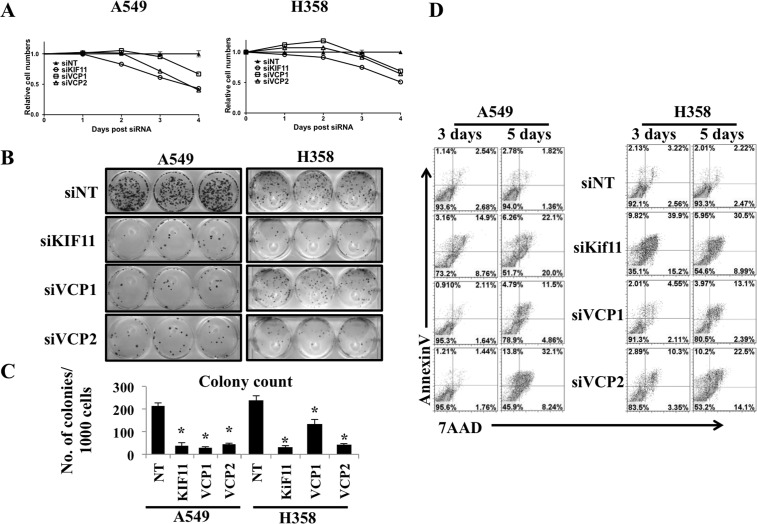
Prolonged VCP loss causes cell death **A.** Analysis of cell viability using Alamar Blue. A549 and H358 cells (500,000) were seeded on 60mm plate and were transfected either with non-targeting siRNA (siNT), positive control Kif11 (siKif11) or with siRNA targeting VCP (siVCP1 and siVCP2). After 24 hrs of transfection, cells were trypsinized and 1000 cells were reseeded in 96 well plates and cell viability was assessed for consecutive 4 days. **B.** VCP loss decreased colony forming ability in A549 and H358 cells, as evaluated by clonogenic assay. Cells were prepared as described in A and after 24 hrs of transfection, cells were trypsinized and 1000 cells were reseeded on 6 well plates in triplicates and the cells were grown for 10 days. Following the 10 days, cells were fixed and stained. **C.** Quantitative evaluation of clonogenic assay. Representative bar graph showing number of colonies formed per 1000 cells seeded. (*P < 0.05). **D.** FACS analysis for cell viability. Cells were transfected as described in A, and 3 days and 5 days following transfections cells were trypsinized wash once with PBS and stained with Annexin and 7AAD in 1X Annexin buffer and FACS analysis for cell death was carried out.

We observed increased in ER stress and its associated UPR following loss of VCP. It is well established that to recover and compensate for ER stress, UPR pathways get activated and excessive ER stress results in apoptosis [6, 7]. Furthermore, recent work from the Vij group demonstrated that sustained inhibition of VCP may be a viable target for killing lung cancer cells [20]. Consistent with these finding we observed decrease in relative cell amounts and colony forming ability of lung adenocarcinoma cells following siRNA mediated loss of VCP for several days (Figures 3A, 3B and 3C). Next we wanted to confirm that the decrease in relative cell numbers following prolonged VCP loss was due to cell death. We transfected A549 and H358 cells either with non-targeting siRNA (siNT), positive control Kif11 siRNA (siKif11) or with siRNA targeting VCP (siVCP1 and siVCP2) and performed FACS analysis 3 and 5 days post transfection (Figure 3D). Interestingly, we found that extensive and prolonged VCP loss causes cell death revealed by Annexin and 7AAD staining. Importantly, we observed that VCP loss affects cell viability and colony forming ability of lung adenocarcinoma cells in concentration dependent manner (Supplemental Figures 1 and 2). These data suggest that sustained loss of VCP results in stress that is not able to be resolved within cells, leading to cell death.

### Eeyarestatin I (EerI) induced ER stress and EMT

Since siRNA mediated loss of VCP leads to cell death, we used pharmacological inhibition of VCP to assess the reversibility of the phenotypes. ERAD inhibitor Eeyarestatin I (EerI) has been shown to inhibit VCP and cause disruption of ER homeostasis. Treatment of A549 and H358 cells with different concentrations of the VCP inhibitor EeyarestatinI (EerI) followed by western blot analysis revealed that treatment with EerI increased ER stress markers including Bip and CHOP as low as 20μM (Supplemental Figure 3A). Furthermore, EerI treatment caused dose dependent cell death of both A549 and H358 cells when EerI was maintained in the culture media for more than 48 hours (Supplemental Figure 4). We also observed what appeared to be dose dependent ubiquitination of key proteins including PERK and IRE1α following treatment with EerI. Importantly, E-cadherin loss and gain of Vimentin only occurred at concentrations of EerI that induced ER stress as measured by activated Bip and Chop (Supplemental Figure 3A). These data suggest that there is an association between ER stress and EMT and suggest that ER stress can induce cells to adopt an EMT-like phenotype. Interestingly, we observed activation of JNK and Akt following treatment with EerI at concentrations sufficient to activate ER stress and induce EMT. This study provides evidence that ER stress can activate both apoptosis and survival pathways (Supplemental Figure 3A). As with treatment of cells with siRNA against VCP, prolonged treatment of cells with EerI also leads to death (Supplemental Figure 5). In addition, we observed increased expression of LC3B, which is activated during autophagy following ER stress [21]. Increased expression of LC3 following treatment with EerI was further confirmed by immunofluorescence staining (Supplemental Figure 3 and 6). EerI-induced EMT in A549 and H358 cells was further confirmed by immunofluorescence staining for the EMT markers E-cadherin and Vimentin (Supplemental Figure 3B). EMT is characterized by increased cell contractility, actin reorganization and formation of projections including membrane protrusions called lamellipodia and filopodia [22-24]. We also observed actin filament reorganization and membrane protrusions following treatment with EerI in A549 and H358 cells (Supplemental Figure 3C). Thus, treatment of cells with a small molecule inhibitor is sufficient to induce both the ER stress and the EMT phenotypes seen when VCP is ablated with siRNA.

### Eeyarestatin induced ER stress, downstream signaling and EMT is reversible

**Figure 4:**
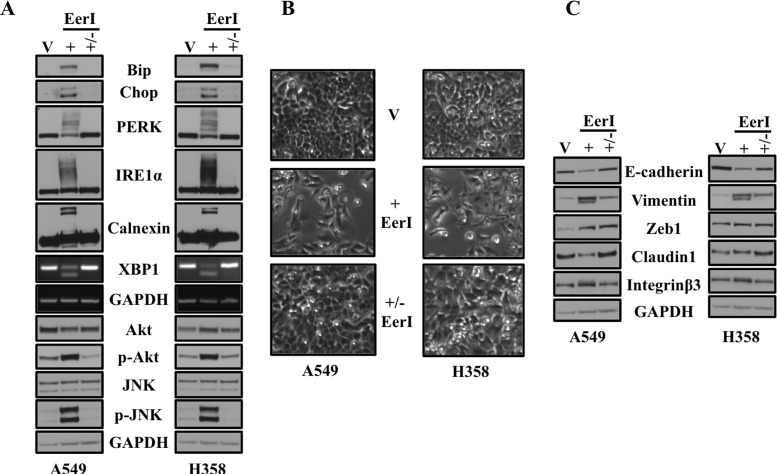
Eeyarestatin induced ER stress, downstream signaling and EMT is reversible **A.** Eeyarestatin induced ER stress and activated downstream signaling cascades are reversible event. A549 and H358 cells were exposed to either vehicle alone (V) or Eeyarestatin (20 μM) (+) for 48 hrs followed by removal of Eeyarestatin by washing the cells and adding fresh media (+/−). After 48 hrs of total exposure and followed by another 48 hrs without Eeyarestatin in medium, cells were harvested and analyzed for protein expression. **B.** Cells were exposed to either vehicle alone or Eeyarestatin as described in A. Cell morphology was assessed after 48 hrs and after removing Eeyarestatin following 48 hrs by phase contrast microscopy before harvesting cells. **C.** Eeyarestatin induced EMT is reversible. Cells were exposed to either vehicle alone or Eeyarestatin as described in A. After 48 hrs of total exposure and followed by another 48 hrs without Eeyarestatin in medium, cells were harvested and analyzed for protein expression. Representative western blot analysis showing expression of proteins involved in EMT.

We observed that sustained inhibition of VCP by either siRNA or EerI activated ER stress and induced EMT in lung adenocarcinoma cells. However, sustained inhibition of VCP by either methodology eventually caused cell death. Therefore, we were interested to see whether ER stress and EMT caused by treatment with EerI were stable or transient phenotypes. For these studies, A549 and H358 cells were treated either with EerI for 48 hrs, followed by another 48 hrs without EerI in medium. As revealed by western bot analysis, treatment with EerI resulted in increased in expression of ER stress response markers including Chop, Bip, PERK, IRE1α and Calnexin (Figure 4A). We also observed splicing of the mRNA encoding XBP-1 following inhibition of VCP. Moreover, further confirming EerI treatment induced UPR, we showed that treatment with EerI activated downstream signaling cascade including Akt and JNK [18] (Figure 4A). Interestingly, we found that increased expression of all ER stress response markers following treatment with EerI return to the basal level once ER stress is removed (Figure 4A). In the present study we also looked for the phenotypic changes by phase contrast microscopy following EerI treatment and after removing ER stress stimulus (Figure 4B). Removal of EerI resulted in reversal of the mesenchymal- and fibroblast-like cell morphology to that of an epithelial appearance by total 96 hrs as revealed by microscopy images (Figure 4B) indicating that VCP loss-induced EMT is reversible event. To further confirm this, we also looked for the expression of different epithelial and mesenchymal markers by harvesting cells at the indicated time points. Interestingly, expression of epithelial markers including E-cadherin and Claudin-1, which was diminished following EerI treatment, was mostly restored following EerI removal. Similarly, expression of mesenchymal markers including Vimentin and Zeb1 which were significantly elevated following EerI treatment was also diminished after EerI removal, however they did not completely return to baseline levels (Figure 4C). In fact, when cells were passaged following removal of EerI the ER stress was completely resolved, however elevated Vimentin persisted for multiple passages, compared to vehicle treated cells (Supplemental Figure 7). The incomplete restoration of EMT markers suggested that this phenotype may be a more stable change within cells.

### Eeyarestatin induces cell migration and cell invasion

**Figure 5:**
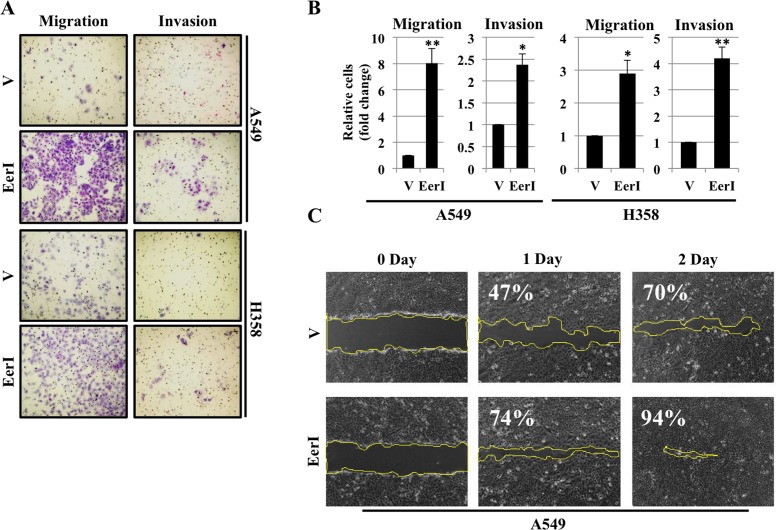
Eeyarestatin induces cell migration and cell invasion **A.** Migration and Invasion assay in A549 and H358 cells. Cells were exposed to either vehicle alone or Eeyarestatin (20 μM) for 48 hrs. After 48 hrs cells were trypsinized, washed once with PBS and seeded into Boyden chambers without (left) or with (right) matrigel. The lower chamber contained media with serum, whereas the upper chamber containing the cells was without serum. 48 hrs later cells on the underside of the membrane were fixed and stained. **B.** Quantification of relative number of cells migrated or invaded through matrigel (*P < 0.05). **C.** Cell migration or wound healing assay in A549 cells. Cells were treated either with vehicle alone or Eeyarestatin for 48 hrs. After 48 hrs cells were washed, trypsinized and reseeded in triplicate. Next day the wound was made by using pipette tip. Cells were examined after wound has been formed and successively for 24hr and 48hr post wound formation and photographed. Quantification of relative percentage wound closure was performed by using ImageJ software.

Increased cell migration and invasion has been shown to be hallmark of EMT [25]. As we observed EerI treatment activated ER stress and induced EMT in lung adenocarcinoma cell lines, we were interested to see whether EerI is capable of altering cell migration and invasiveness. We observed incomplete restoration of EMT markers following removal of EerI, so we hypothesized that this incomplete restoration of EMT may be associated with increased migration and invasion. To this end, we performed ‘Boyden chamber’ cell migration and invasion assay using A549 and H358 cells that were pre-treated with EerI for 48 hours. Interestingly, cells treated with EerI acquired more migratory and invasive phenotype as determined by the number of cells that migrated and invaded through matrigel compared with cells treated with vehicle alone (Figures 5A and 5B). Our findings are consistent with the previous observations by Levina et al. where by using lung cancer cell line H460 they showed that lung tumor cells survived after chemotherapeutic drug treatment, carries all the properties of cancer tumor cells and have more clonogenic and invasive properties [26]. Treatment with EerI induces cell migration was further confirmed by using *in vitro* wound healing assay in A549 cells. After 48hrs of treatment either with vehicle alone or with EerI, cells were washed with PBS, trypsinized and replated. Next day a “wound” was formed and cells were examined after 24 hrs and 48hrs post wound formation. Treatment with EerI showed nearly complete healing of the wound after 48 hrs compared with cells exposed to vehicle alone (Figure 5C), which had comparatively fewer cells migrated into the gap suggesting that EerI treatment enhances tumor cell migration. Furthermore, we observed increased expression of key proteins involved in cell cycle regulation including RhoA, Rac1 and Cdc42 following loss of VCP (Supplemental Figure 8).

As we observed ER stress and EMT are closely associated following VCP inhibition, we were interested to ensure that cell migration and invasion was not altered using a concentration of EerI that was not capable of activating an ER stress response, and thus did not increase an EMT-like program. To accomplish this we repeated the same set of experiments described above, but instead of using 20 μM, we pre-treated the cells with 5 μM EerI. Cell migration and invasion were not altered under these conditions (Supplemental Figures 9 and Figure 10).

### Akt or Src kinase inhibitors block ER stress and EMT in lung adenocarcinoma cells

**Figure 6:**
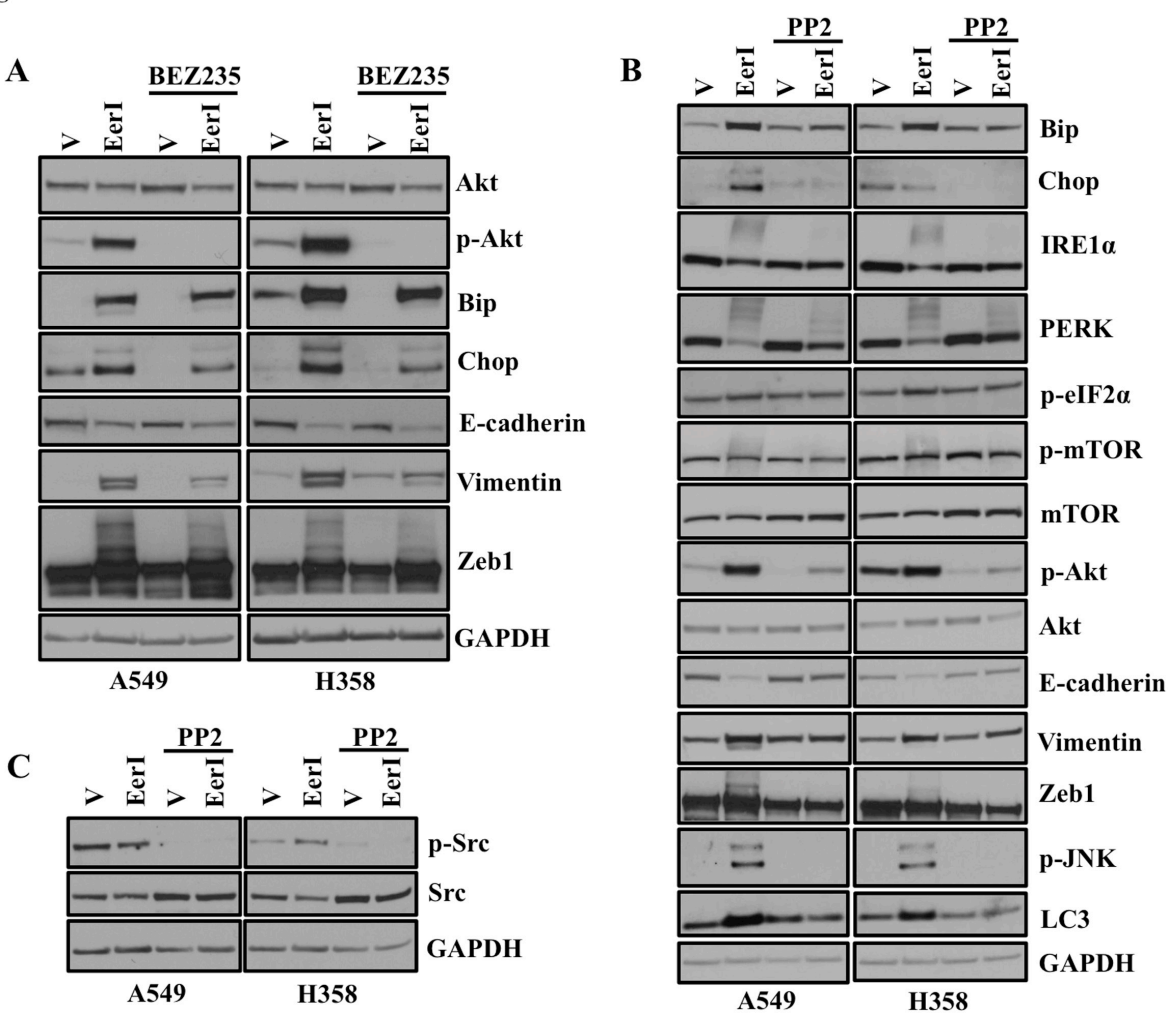
Akt or Src kinase inhibitors block ER stress and EMT in lung adenocarcinoma cells **A.** Treatment with BEZ235 partially blocks ER stress and EMT in lung adenocarcinoma cells. A549 and H358 cells were exposed to either vehicle alone or Eeyarestatin (20μM) for 48 hrs and treated with BEZ235 (1 μM) for 24 hrs before harvesting. Representative western blot analysis on the (left) showing expression of proteins involved in ER stress and EMT. **B.** PP2 blocks ER stress and its associated EMT in lung adenocarcinoma cells. A549 and H358 cells were pretreated with PP2 (20 μM) and then cotreated with either vehicle alone or Eeyarestatin (20 μM) for 48 hrs. Representative western blot analysis on the (right) showing expression of proteins involved in ER stress and EMT. **C.** Representative western blot analysis showing the on target effect of PP2.

Akt has been shown to be involved in cellular proliferation, survival and EMT [27, 28]. As we observed Akt activation following inhibition of VCP with EerI (Supplemental Figure 3A), we were interested to see whether inhibiting Akt function could block EMT. Interestingly, Akt inhibition with BEZ235 partially blocks ER stress and its associated EMT in lung adenocarcinoma cells induced by EerI, as revealed by western blot analysis (Figure 6A). Src family kinases are overexpressed and activated in a variety of tumors including breast, pancreatic, ovarian and head and neck [29]. Therefore, there is vast amount of interest in development and use of Src kinase inhibitors as potential anticancer therapeutics. In the present study we observed activation of Akt and JNK following ER stress (Supplemental Figure 3A). Thus, we were interested to see whether inhibiting Src kinases, by using well established inhibitor PP2, could block ER stress and its associated downstream EMT. Interestingly, we found that increased expression of all ER stress response markers including Bip, Chop, IRE1α, PERK and p-eIF2α following treatment with EerI return to the basal level following treatment with Src kinase inhibitor PP2 (Figure 6B). We also looked for the expression of different epithelial and mesenchymal markers by harvesting cells at same time points. Interestingly, expression of epithelial marker E-cadherin, which was diminished following EerI treatment, was restored following PP2 treatment. Similarly, expression of mesenchymal markers including Vimentin and Zeb1 were significantly increased following EerI treatment and returned to basal level after PP2 treatment (Figure 6B) further providing evidence that PP2 blocks ER stress and its associated EMT in lung adenocarcinoma cells. We were also able to rescue the Akt and JNK activation following treatment with EerI by PP2 indicating Src kinase inhibitors PP2 can be used to block the ER stress and its associated EMT in lung adenocarcinoma cells.

### VCP expression, ER stress and EMT in primary human lung adenocarcinomas

**Figure 7:**
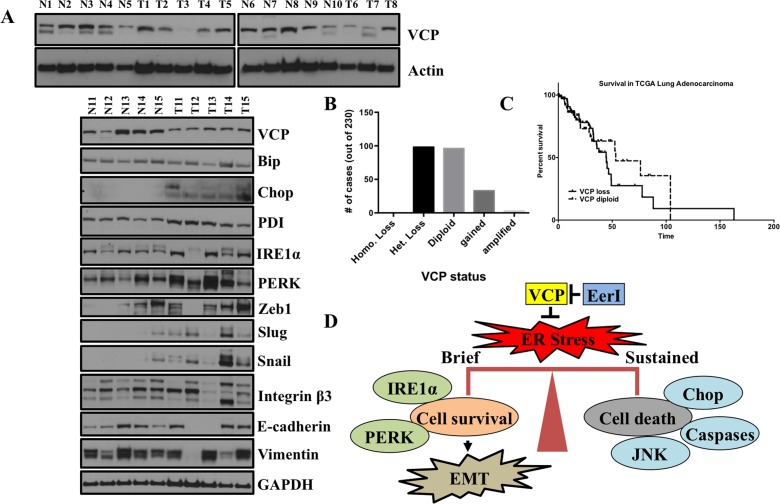
VCP is underexpressed in primary lung adenocarcinoma patients, ER stress and EMT markers are co-expressed in primary lung tumors from patients compared to adjacent matched tissues **A.** VCP is under expressed in primary lung adenocarcinoma patients. Representative western blot showing VCP expression and different proteins involved in ER stress and EMT in primary lung adenocarcinomas vs normal lung adjacent tissues. **B.** and **C.** Analysis of TCGA for VCP status in lung adenocarcinoma patients and their survival. Out of 230 lung adenocarcinoma patients analyzed mostly patients have either diploid or heterozygous loss. TCGA analysis of lung adenocarcinoma indicating patients with VCP diploid status have shorter survival. **D.** Schematic depicting how VCP regulates the balance between ER stress-induced EMT and ER-stress induced cell death.

ER stress and its associated UPR have been shown to be associated with different diseases including lung cancer [30]. We observed ER stress after loss of VCP function, which was followed by EMT in lung adenocarcinoma cell lines. Thus, we were interested to see the expression level of VCP in lung adenocarcinoma patients compared with matched normal adjacent tissues. Western blot analysis revealed that VCP protein is underexpressed in the vast majority primary lung adenocarcinomas, when compared to normal adjacent tissue (Figure 7A). Moreover, analysis of TCGA data revealed that the VCP locus is lost in nearly 50% of lung adenocarcinoma samples (Figure 7B). However, analysis of patient survival did not show a significant alteration in overall outcome when patients were stratified by VCP locus loss (Figure 7C).

Since we observed induction of ER stress and EMT following loss of VCP function *in vitro* we were interested to see whether there was a similar correlation *in vivo*. For that, we again compared the expression of ER stress and EMT markers in matched normal vs adjacent tumors. Interestingly, we find that expression of ER stress markers including Bip, Chop, IRE1α, PERK and PDI were significantly elevated in matched lung tumors from adenocarcinoma patients compared to normal adjacent tissues (Figure 7A). Also we observed elevated expression level of mesenchymal markers including Snail, Zeb1 and Vimentin in matched lung tumors from adenocarcinoma patients compared to normal adjacent tissues (Figure 7A). These data suggest that VCP function may be important in maintaining the pathophysiology of ER and its associated EMT and may be useful for implementing better therapeutic and predictive strategies.

## DISCUSSION

The ER in cells of eukaryotic organisms is involved in a vast diversity of functions including proper protein folding [31, 32]. Disturbances in ER homeostasis lead to accumulation of misfolded proteins in the ER, which in turn leads to ER stress and the unfolded protein response (UPR). During the initial phase, in order to compensate for the UPR, signaling events induce the expression of various genes to remove or aid in properly re-folding of the misfolded proteins [33]. Valosin-containing protein (VCP), also called p97 or cdc48 in yeast, is known to be involved in wide variety of functions including endoplasmic reticulum-associated degradation (ERAD) [34, 35] and is responsible for maintaining the integrity of ER. VCP loss results in ER stress [36]. Consistent, in our studies involving lung adenocarcinoma cell lines, we observed that loss of VCP resulted in significant increase in ER stress and associated UPR, as evident from increased expression of different proteins including Bip, Chop and phosphorylation of eukaryotic initiation factor 2 (eIF2)-α.

Recently, studies suggested that ER stress induces EMT in different biological systems [37, 16, 17]. Furthermore, the level of ER stress to which cells are subjected appears to be a critical determinant of whether cells undergo apoptosis or EMT [15]. In the present study, we sought to study the role of VCP in the induction of EMT either through genetic loss or by using a chemical modulator of VCP. Interestingly, we found that VCP loss by either methodology activated ER stress and induced EMT in lung adenocarcinoma cells. Growing evidences suggests that extensive ER stress resulted in apoptosis [6, 38-40]. Therefore, we were interested to study the effect of brief and sustained loss of VCP towards cell viability. Interestingly, we found that with brief ER stress, EMT is activated and with sustained loss of VCP cells eventually die.

Using VCP inhibitor EerI, we tried to optimize the concentration at which the EerI activated low levels of ER stress, but not EMT. We were not able to find such a concentration and rather we noticed that ER stress was either present or absent. Likewise, any concentrations of EerI that did induce UPR, markers of EMT were also induced. Furthermore, we have demonstrated that ER stress, through loss of VCP, induced EMT in lung adenocarcinoma cell lines, suggesting a potential mechanism whereby ER stress may contribute to cancer progression through regulating EMT.

During ER stress-induced EMT, epithelial cells undergo phenotypic changes, which are characterized by the loss of epithelial markers and the gain of mesenchymal markers; these phenotypic changes are associated with cancer progression [41, 42] however so far it is poorly understood whether ER stress induced EMT is reversible event. To address this void in our understanding, we provided ER stress and removed it after some instance. Interestingly, we observed that ER stress activates EMT at initial phase and once VCP function is restored the cells quickly resolve the ER stress and survive indefinitely. Interestingly, however, the reversion back to an epithelial-like state was more delayed, such that after restoration of VCP activity, by removal of EerI, cells still showed EMT characteristics and retained the expression of some of the key proteins involved in EMT including Vimentin and Zeb1 indicating that EMT downstream of ER stress is a longer term effect, although after several days this phenotype is also capable of reverting back to pre-treatment levels. Our study indicates that there exists balance between cell survival and cell death (Figure 7D). The duration of ER stress decides whether cells will survive and activate EMT or they will enter apoptosis [6]. It has been shown that during EMT, epithelial cells undergo various dramatic changes. Epithelial cells lose the expression of tight adherents-junction proteins such as E-cadherin and Claudin1 and become more migratory and invasive [43-46]. Consistent with these findings, our study involving lung adenocarcinoma cell lines we showed that inhibition of VCP by EerI induces ER stress and activates EMT by becoming more migratory and invasive.

Recent interest has been in the development of chemicals or drugs that can target the proteins that are more abundant in cancer development pathways and typically involved in cell growth and survival. Akt activity has been shown to be associated with tumor cell survival, proliferation, and invasiveness [47, 48]. Consistent with these finding we observed activation of Akt following treatment with EerI and more interestingly, we observed inhibiting Akt can partially blocks ER stress and its associated EMT in lung adenocarcinoma cells providing further evidence that Akt may contribute towards EMT by activating proliferation and survival pathways in cellular system. Furthermore, our study showed that the Src kinase inhibitor PP2 completely blocks ER stress and EMT induced by VCP inhibition in lung adenocarcinoma cells.

Extent of ER stress has been shown to be critical a determinant of life and death decision. Herein, we showed that VCP is underexpressed in primary matched lung tumors vs normal adjacent tissues. More interestingly, we observed that ER stress and EMT markers are co-expressed in matched lung tumors vs normal adjacent tissues. Furthermore, we find that the VCP locus is lost in nearly 50% of lung adenocarcinoma patients, suggesting that VCP function may be critical in lung cancer progression.

## CONCLUSION

The present study demonstrates that ER stress caused by disruption of VCP is associated with EMT in lung adenocarcinoma cells (Figure 7D). These findings suggest an important and direct role for drug induced and chronic ER stress in lung cancer progression and metastasis. Furthermore, a complete understanding for how activation of ER stress induces EMT will be useful for the development of safer and more effective anticancer strategies.

## MATERIALS AND METHODS

### Human primary tumor and adjacent normal lung tissue samples

Human primary tumor and adjacent normal lung tissue samples were obtained from tissue bio-repository facility of James Graham Brown Cancer Center, at University of Louisville. Local IRB committee of the University of Louisville approved the proposed human study. Tissues were lysed in CEB lysis buffer. Protein was estimated and equal amount of protein was used for studying expression of proteins involved in ER stress and EMT.

### Antibodies used for study

VCP #2649, Bip #3183, CHOP #2895, eIF2α #9722 and p-eIF2α #9721, IRE1α #3294, PERK #5683, JNK #9258, p-JNK 4668, p38 #8690, P-p38 #4511, LC3 #3868, Cleaved Caspase3 #9664, Calnexin #2679, PDI #3501, E-cadherin #3195, Vimentin #5741, Zeb1 #3396, Claudin1 #4933, Snail #3879, Slug #9585, Integrin β3 #4702, Akt #9272, P-Akt #9271, mTOR #2972, P-mTOR #2971 (Cell Signaling Technologies Inc. Danvers, MA 01923); GAPDH #FL335 (Santa Cruz); Alexa Fluor 488 goat anti-rabbit IgG #A11034 and Alexa Fluor 546 goat anti-rabbit IgG #A11010 (Molecular Probes, Invitrogen detection technologies, Eugene, OR. USA); Alexa Fluor 488 rabbit anti-mouse #A-11059 and Alexa Fluor 568 Phalloidin #A12380 (Life technologies Eugene, OR. USA). APC Annexin V #550475, 7AAD #51-68981E (BD Biosciences, San Jose, CA 95131).

### Cell culture and siRNA transfection and protein analysis

Human adenocarcinoma cell lines A549, H358 and human immortalized small airway epithelial cell line (HPLD-1) were purchased from American Type Culture Collection (ATCC, Rockville, MD, USA) and cultured in RPMI medium supplemented with 10% fetal bovine serum (Invitrogen, Carlsbad, CA, USA) and 1% antibiotic/antimycotic (Sigma, St Louis, MO, USA). The cell lines were routinely subcultured every 3–5 days. All siRNA transfections were performed using Dharmafect1 # T-2001-03 (Thermo Fisher Scientific Inc, Pittsburgh, PA, USA) as per manufacturer's protocol. After total 72 hrs of transfection cells were harvested in CEB lysis buffer # FNN0011 (Invitrogen, Life technologies, Grand Island, NY, 14072). Protein was quantitated by using Pierce's BCA Protein Assay Reagent Kit (# 23227) from Pierce Biotechnology, Rockford, IL, USA as per manufacturer's protocol (for more detail regarding siRNA sequences used for study, see Supplemental Methods).

### Immunofluorescence staining

Immunofluorescence was carried out as described previously [49]. Briefly, after total 24 hrs of transfection either with non-targeting siRNA (siNT) or with siRNAs targeting VCP (siVCP1 and siVCP2), cells were trypsinized and plated in chamber slides for immunofluorescence staining. Similarly, cells were also plated on chamber slides and treated with either vehicle alone or with 20μM EerI for 48 hrs in medium. After 72 hrs of transfection or with 48hrs EerI treatment, cells were fixed with 4.0% paraformaldehyde in PBS for 30 min and then permeabilized with 0.2% Triton X-100 for 15 min at room temperature. Cells were rinsed twice with PBS, and then incubated for 1-2 hrs with anti-Vimentin antibody (at a dilution of 1:1000) or anti-E-cadherin antibody (at a dilution of 1:1000) or anti-LC3 antibody (at a dilution of 1:1000). After successive washes, cells were then incubated with Alexa Fluor 488 rabbit anti-mouse IgG #A11059 (Life technologies Eugene, OR. USA) or Alexa Fluor 488 goat anti-rabbit IgG #A11034 or Alexa Fluor 546 goat anti-rabbit IgG #A11010 (Molecular Probes, Invitrogen detection technologies, Eugene, OR. USA). After incubation with secondary antibody for 45 min, cells were rinsed with PBS and incubated with Alexa Fluor 568 Phalloidin #A12380 (Life technologies Eugene, OR. USA) at the dilution of 1:1000 to stain actin filaments. After successive washing with washing buffer, nuclei were counterstained with DAPI (diluted 1:000) for 10 min at room temperature followed by three washes (5–10 min each) with washing buffer. The cells were then mounted with polymount antifade solution (Sigma) and observed under MRC 600 confocal laser scanning microscope (Bio-Rad). Fluorescence staining for E-cadherin, Vimentin and LC3 in A549 and H358 cells following EerI drug treatments was carried out in the similar fashion as described earlier.

### RNA extraction, cDNA synthesis, and RT-PCR

After 48 hrs of treatment either with vehicle alone or with EerI and further 48 hrs without EerI in medium and similarly, after 72 hrs of transfection of A549 and H358 cells either with non-targeting siRNA (siNT) or with siRNAs targeting VCP (siVCP1 and siVCP2), cells were harvested and total RNA was purified using TRI reagent (#9424) (Sigma-Aldrich, Inc. St. Louis, MO. USA), according to the manufacturer's protocol (for more detail regarding cDNA synthesis and RT-PCR see Supplemental Methods).

### Oligo sequences of XBP-1 used for study

Oligos for XBP-1 used for the study were ordered from Invitrogen, Life technologies, Grand Island, NY, 14072 USA.

XBP-1 FP: CCTGGTTGCTGAAGAGGAGG

XBP-1 RP: CCATGGGGAGATGTTCTGGAG

## SUPPLEEMENTARY MATERIAL AND METHODS AND FIGURES


