# Supplementary material for: Regulation of VCP/p97 demonstrates the critical balance between cell death and epithelial-mesenchymal transition (EMT) downstream of ER stress
Source: Oncotarget. 2015 Apr 23;6(19):17725–37. doi: 10.18632/oncotarget.3918 (PMC4627341; doi:10.18632/oncotarget.3918)
Supplement: Supplementary file 1 [file oncotarget-06-17725-s001.pdf]

Regulation of VCP/p97 demonstrates the critical balance between cell death and epithelial-mesenchymal transition (EMT) downstream of ER stress

Supplementary Material and Methods

Western blot analysis

Cells were lysed in Cell Extraction buffer (CEB #FNN0011), Invitrogen Corporation, Camarillo, CA. USA supplemented with Complete Mini Protease Inhibitor Tablets and PhosSTOP EASYpack Phosphatase Inhibitor Cocktail Tablets (Roche Molecular Biochemical, Indianapolis, IN, USA) wherever required. Protein was quantitated by using Pierce BCA protein assay kit, from Pierce Biotechnology (Rockford, IL, USA). Equal amount of protein (40 µg) from each sample was added to SDS loading buffer, boiled, and resolved on a 4-12% SDS–PAGE gel and transferred onto a membrane. Blots were probed with different antibodies and immunoreactive proteins were visualized using the SuperSignal West Femto Maximum Sensitivity Substrate (# PI34095) from Fisher Scientific (Pittsburgh, PA 15275 USA), according to manufacturer's instruction. The membrane was stripped by using western blot stripping reagent (BioRad, Hercules, CA, USA) and reprobed with GAPDH antibody to normalize the variation in loading of samples.

Cell Viability and Clonogenic assay

A549 and H358 cells were cultured in 60 mm culture plates. After 24 hrs of transfection either with non-targeting siRNA (siNT), positive control Kif11 siRNA (siKif11) or with siRNA targeting VCP (siVCP1 and siVCP2), cells were trypsinized, counted and 1000 cells were reseeded per well in 96 well plates. Cell viability was analyzed for successive 4 days using Alamar blue (#DAL1100 Invitrogen detection technologies, Eugene, OR. USA). At the same

time following transfection, cells were reseeded in 6 well plates in triplicate for each transfection. Cells were allowed to grow on 6 well plates for 10 days. Cells were supplemented with fresh media after every 2-3 days. After 10 days, colonies formed were washed once with PBS, fixed with 70% ethanol and stained with 0.25% coomassie brilliant blue stain R (#B 7920 Sigma-Aldrich, Inc. St. Louis, MO. USA) and photographed.

RNA Extraction, cDNA Synthesis, and RT-PCR

After 48 hrs of treatment either with vehicle alone or with EerI and further 48 hrs without EerI in medium and similarly, after 72 hrs of transfection of A549 and H358 cells either with non-targeting siRNA (siNT) or with siRNAs targeting VCP (siVCP1 and siVCP2), cells were harvested and total RNA was purified using TRI reagent (#9424) (Sigma-Aldrich, Inc. St. Louis, MO. USA), according to the manufacturer's protocol. The quality of the isolated RNA was assessed using the Bioanalyzer 2100 (Agilent Technologies, Inc., Wilmington, DE) and quantification was performed using a Nanodrop 1000 (Thermo Scientific, Wilmington, DE). cDNA was synthesized with High Capacity RNA-to-cDNA Kit from Applied Biosystems # 4387406 (Applied Biosystem, Foster City, USA). The PCR amplification was performed by using One Taq^{TM} Hot Start 2X Master Mix #M0484S (New England Biolabs, Ipswich, MA), with 0.2 µM of each primer, 10 µl of 2X master mix and 1 µl of cDNA template, in a final reaction volume of 20 µl using the following cycle parameters: enzyme activation at 94^{0}C for 30 s; 35 cycles of 94^{0}C for 15 s, 60^{0}C for 30 s and 68^{0}C for 1 min, and 5 min of final extension at 68^{0}C. PCR products were directly analyzed by separation on 3% agarose gels. All experiments were repeated three times using three independent preparations of cDNA.

siRNA sequences used for study

All siRNAs used for study were ordered from Thermo Fisher Scientific Biosciences Inc. Lafayette, CO 80026, USA.

Non targeting siRNA (siNT): UAAGGCUAUGAAGAGAUACAA

VCP siRNA (siVCP1): GUAAUCUCUUCGAGGUAUA

(siVCP2): CAAAUUGGCUGGUGAGUCU

KIF11 pool siRNA (siKIF11) Cat. No. L-003317-00-0010

FACS analysis for apoptosis

FACS analysis was performed by using FACScan Flow Cytometer from Cytek Development, Fremont, CA. USA. Briefly, after 72 hrs and 120 hrs of post transfection of A549 and H358 cells with either siRNA targeting VCP (siVCP1 and siVCP2) or with positive control kif11 and with non-targeting siRNA (siNT), cells were trypsinized and washed once with PBS. After washing cells were suspended in 1X Annexin buffer along with Annexin and 7AAD and incubated at 4^0C for 20 min in dark. The cells were washed once again with PBS and were resuspended for analysis on a FACScan Flow Cytometer and analyzed with FlowJo software.

Drug treatments

A549 and H358 cells either treated with vehicle alone or with indicated concentrations of Eeyarestatin (EerI) (Cayman) for 48 hrs. Cells were then trypsinized and used for further studies or then harvested and total lysates were used for expression studies. In case of Akt inhibitor BEZ235 (Cayman) treatment, cells were exposed to either vehicle alone or Eeyarestatin I (20µM) for 48 hrs and treated with BEZ235 (1µM) for 24 hrs before harvesting. For Src kinase inhibitor treatment, cells were pretreated with PP2 (Cayman) (20µM) and then cotreated with

either vehicle alone or Eeyarestatin I (20µM) for 48 hrs. Cells were then harvested and total lysates were used for expression studies.

Cell migration assay or Wound healing assay

A549 cells were plated 100mm plate and exposed to either vehicle alone or EerI for 48 hrs. Cells were washed with PBS, trypsinized, counted and replated in triplicate in 6-well plate. Next day, wound were made using the pipette tip following replacement with fresh media. Cells were examined successively after 24 hrs and 48 hrs of wound formation and photographed. Average of percentage wound closure of randomly selected three areas was calculated by using ImageJ (NIH, USA) software.

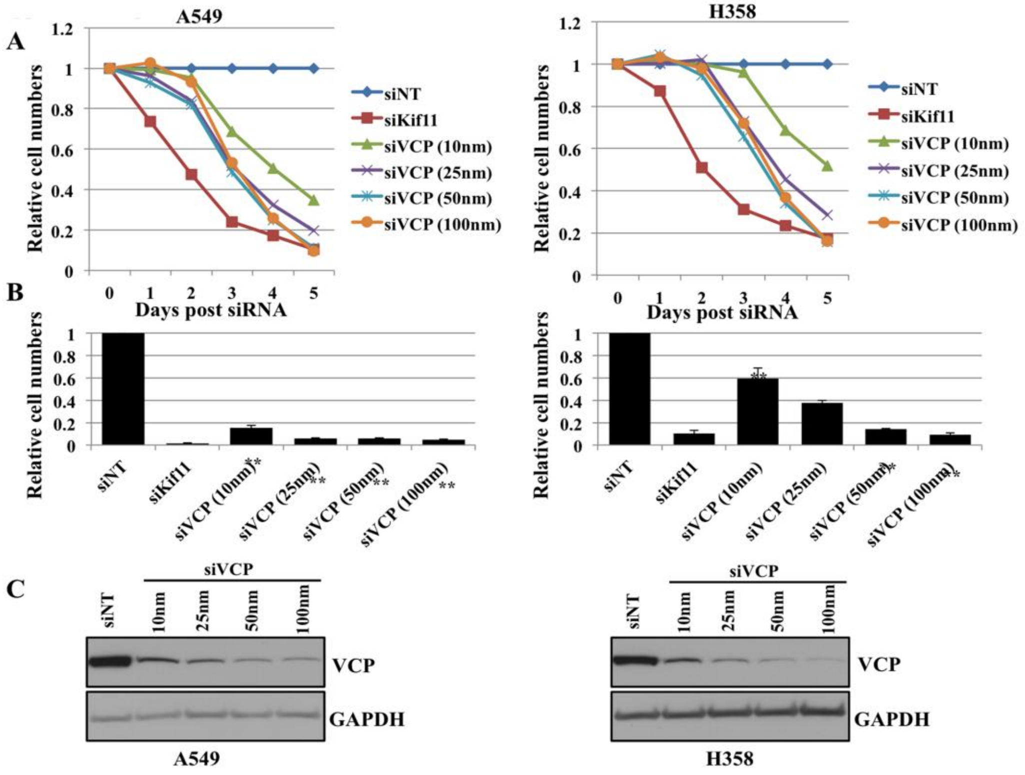

Supplemental Figure 1: Sustained ER stress following VCP loss leads to cell death. (A) Analysis of cell viability using Alamar Blue. A549 and H358 cells (500,000) were seeded on 60mm plate and were transfected either non-targeting siRNA (siNT), positive control Kif11 (siKif11) or with different siRNA concentrations targeting VCP. After 24 hrs of transfection, cells were trypsinized and 1000 cells were reseeded on 96 well plates and cell viability was assessed for consecutive 5 days. (B) Analysis of cell viability using Alamar Blue at 10th day. Cells were prepared as described in A and after 24 hrs of transfection, cells were trypsinized and 1000 cells were reseeded on 6 well plates in triplicate and the cells were grown for 10 days. Following the 10 days, cell viability was assessed at 10th day. Representative bar graph showing cell viability following sustained VCP loss. (**P < 0.005). (C) VCP Knockdown confirmation by western blot analysis. Cells were prepared as described in A and 72 hrs of post transfection, cells were cells were harvested and analyzed for protein expression.

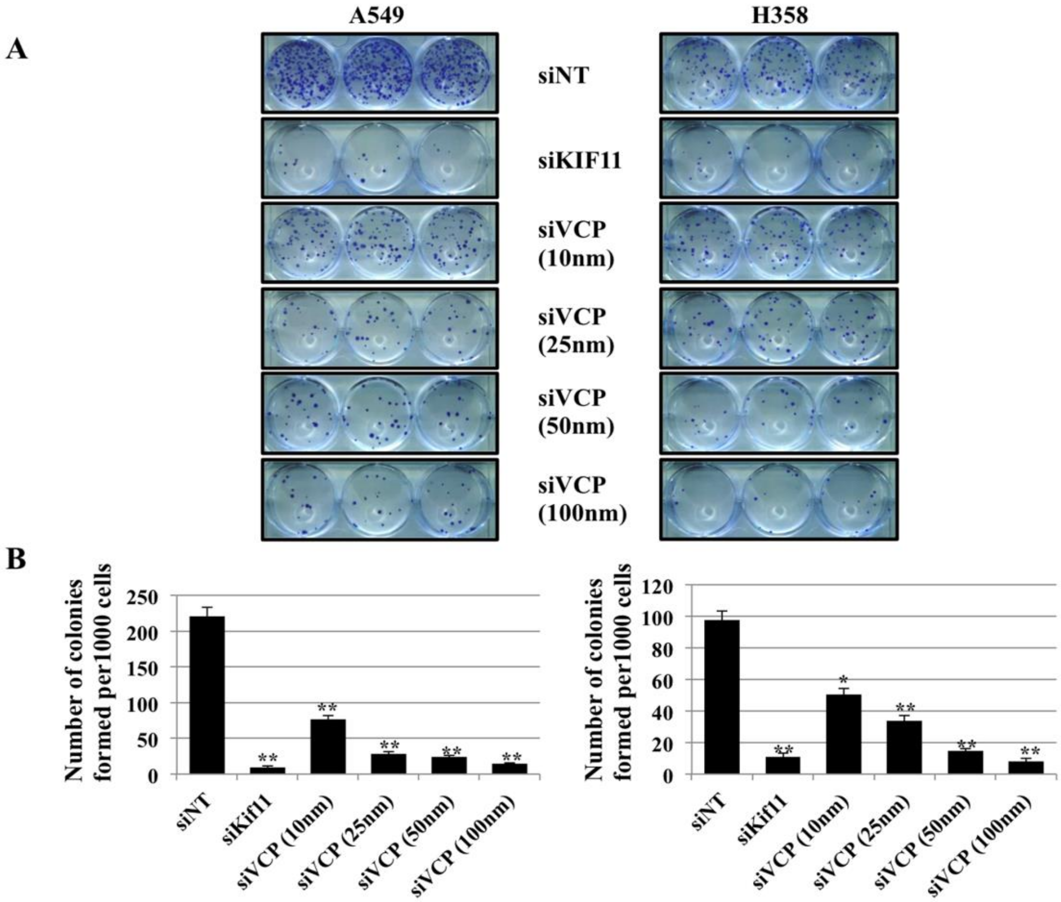

Supplemental Figure 2: Sustained ER stress following VCP loss leads to decrease colony forming ability of lung adenocarcinoma cells. (A) VCP loss decreased colony forming ability in A549 and H358 cells, as evaluated by clonogenic assay. A549 and H358 cells (500,000) were seeded on 60mm plate and were transfected with either non-targeting siRNA (siNT), positive control Kif11 (siKif11) or with different siRNA concentrations targeting VCP. After 24 hrs of transfection, cells were trypsinized and 1000 cells were reseeded on 6 well plates in triplicate and the cells were grown for 10 days. Following the 10 days, cells were washed once with PBS, fixed with 70% ethanol, and stained with coomassie blue stain. (B) Quantitative evaluation of clonogenic assay. Representative bar graph showing number of colonies formed per 1000 cells seeded. (*P < 0.05, **P < 0.005).

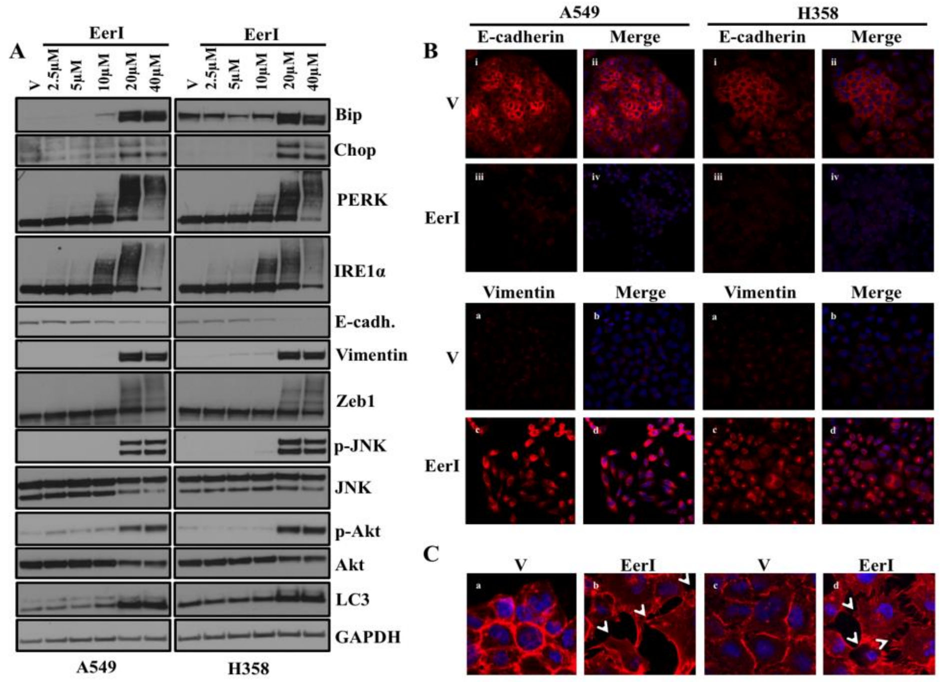

Supplemental Figure 3: Eeyarestatin (EerI) induced ER stress and EMT. (A) Eeyarestatin induced ER stress and EMT in lung adenocarcinoma cells. A549 and H358 cells were exposed to either vehicle alone or with indicated concentrations of Eeyarestatin for 48 hrs. Representative western blot analysis on the (left) showing expression of proteins involved in ER stress and EMT. (B) Fluorescence staining of E-cadherin and Vimentin in A549 and H358 cells. Cells were plated on chamber slides and after 48 hrs of treatment either with vehicle alone or with eeyarestatin, cells were fixed stained for E-cadherin and Vimentin. i and iii: E-cadherin was detected using Alexa Fluor 546 goat anti-rabbit IgG (red). ii and iv: overlay of respective E-cadherin with DAPI counter stain. a and c: Vimentin was detected using Alexa Fluor 546 goat anti-rabbit IgG (red). b and d: overlay of respective Vimentin with DAPI counter stain. (C) Cells were prepared as described in A and F-actin was detected with Alexa Fluor 568 Phalloidin (red). Re-organization of actin cytoskeleton through destruction and cellular protrusion formation is indicated by arrows.

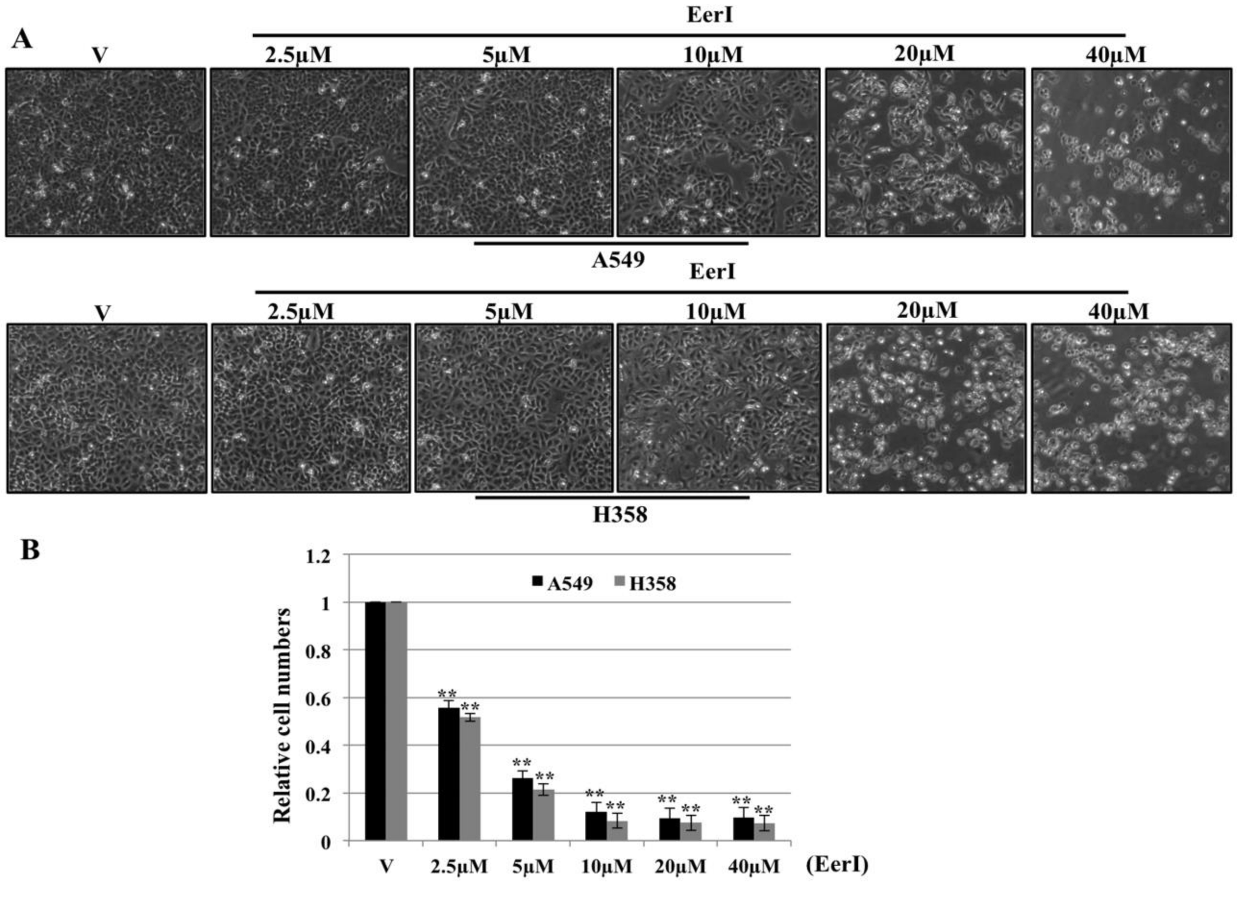

Supplemental Figure 4: Prolonged eeyarestatin I (EerI) treatment induces cell death. (A) A549 and H358 cells were either treated with vehicle alone or with indicated concentrations of EerI for 48 hrs. Cell morphology was assessed after 48 hrs of treatment by phase contrast microscopy. (B) Analysis of cell viability using Alamar Blue. 3000 cells were plated on 96 well plate followed by treatment with either vehicle alone or Eeyarestatin. After 48 hrs of treatment cell viability was assessed by Alamar blue. Representative bar graph showing cell viability following Eeyarestatin treatment. (**$P < 0.005$).

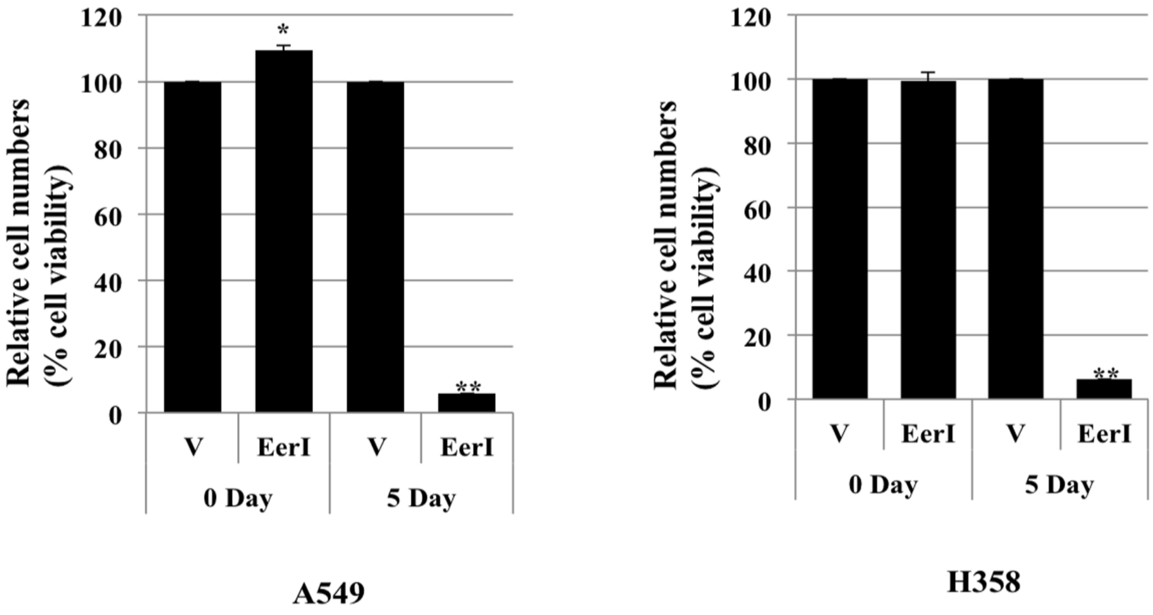

Supplemental Figure 5: Sustained ER stress following EerI treatment leads to cell death. Analysis of cell viability using Alamar Blue. A549 and H358 cells (1,000) were seeded on 96 well plates and treated with either vehicle alone or with EerI (20µm) for 5 days. Following treatment cell viability was assessed. Representative bar graph showing cell viability following sustained ER stress. (*P < 0.05, **P < 0.005).

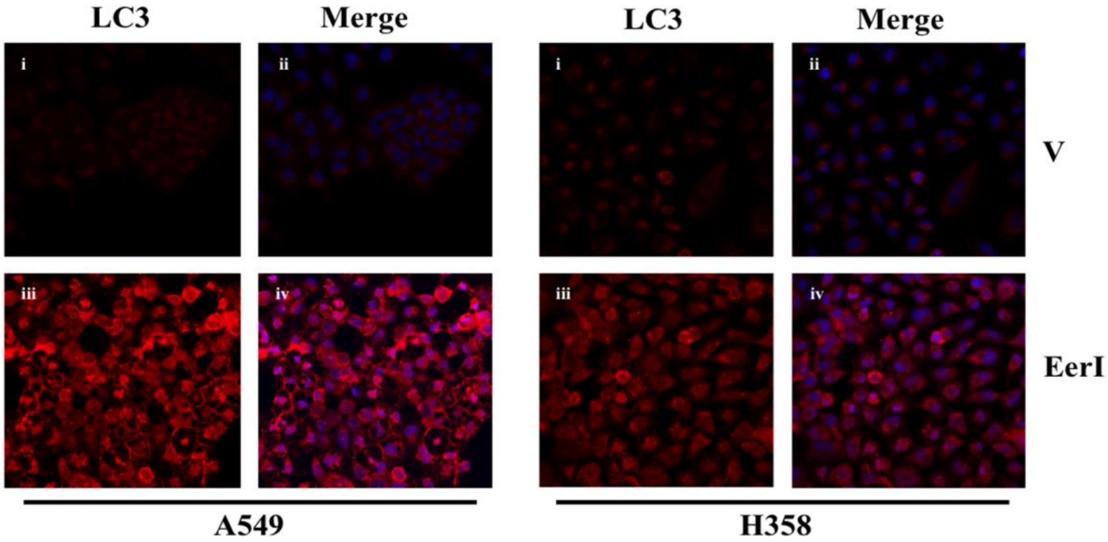

Supplemental Figure 6: Eeyarestatin induced autophagy as revealed by turnover of LC3B. Fluorescence staining of LC3 in A549 and H358 cells. Cells were plated on chamber slides and after 48 hrs of treatment either with vehicle alone or with Eeyarestatin, cells were fixed stained for LC3. i and iii: LC3 was detected using Alexa Fluor 546 goat anti-rabbit IgG (red). ii and iv: overlay of respective LC3 with DAPI counter stain.

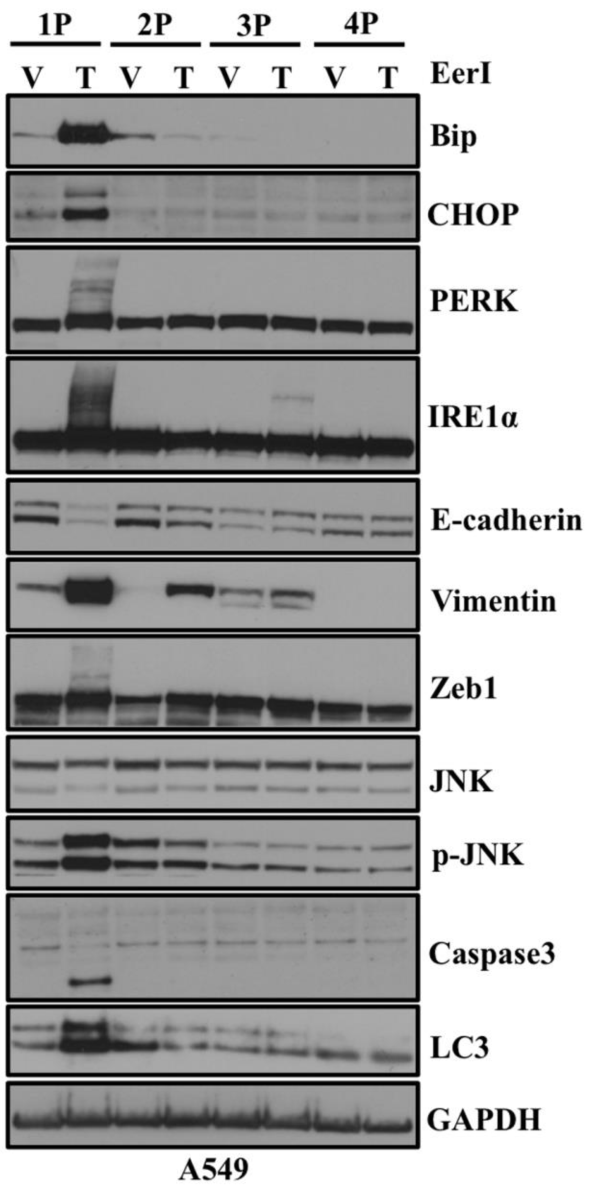

Supplemental Figure 7: Determiation of the stability of EMT-like phenotype. A549 cells were either treated with vehicle alone or Eeyarestatin (20µM) for 48 hrs and were successively passaged following removal of EerI. Cells were harvested at the indicated time points. Representative western blot analysis showing expression of proteins involved in ER stress and EMT.

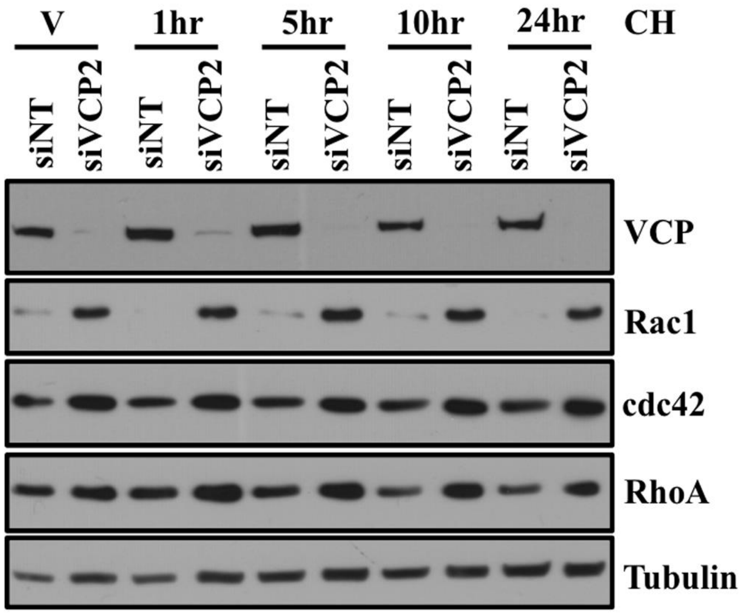

Supplemental Figure 8: VCP loss increases proteins involved in cell motility. A549 cells were transfected with either non-targeting (siNT) siRNA or siRNA targeting VCP (siVCP). Cells were exposed to either vehicle alone or CH for indicated time before total 72 hrs of transfection. Cells were harvested at the indicated time points. Representative western blot analysis showing expression of proteins involved in cell cycle regulation.

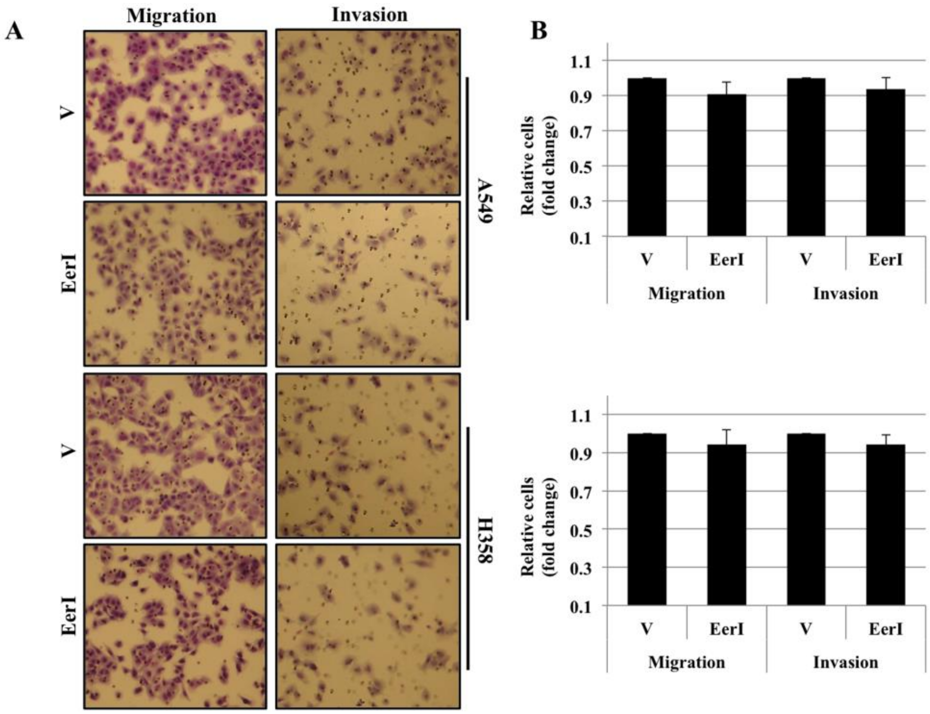

Supplemental Figure 9: Eeyarestatin (5μM) does not alter cell migration and cell invasion when ER stress is not induced. (A) Migration and Invasion assay in A549 and H358 cells. Cells were exposed to either vehicle alone or Eeyarestatin (5μM) for 48 hrs. After 48 hrs cells were trypsinized, washed once with PBS and seeded into Boyden chambers without (left) or with (right) matrigel. The lower chamber contained media with serum, whereas the upper chamber containing the cells was without serum. 48 hrs later cells on the underside of the membrane were fixed and stained. (B) Quantification of relative number of cells migrated or invaded through matrigel (*P < 0.05).

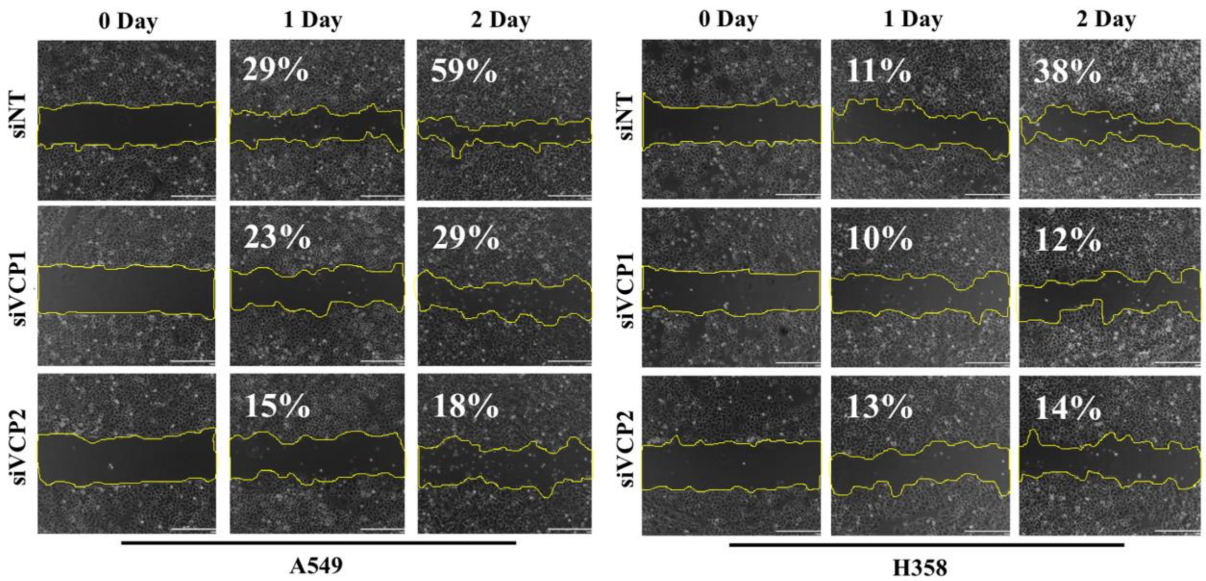

Supplemental Figure 10: Sustained loss of VCP does not alter cell migration. Cell migration or wound healing assay in A549 and H358 cells. A549 and H358 cells (300,000) were seeded on each well of 6 well plates and were transfected either with non-targeting siRNA (siNT), or with siRNA targeting VCP (siVCP1 and siVCP2). After 24 hrs of transfection, wound was made by using pipette tip. Cells were examined after wound has been formed and successively for 24hr and 48hr post wound formation and photographed. Quantification of relative percentage wound closure was performed by using ImageJ software.
